# Traditional Chinese Medicine for childhood obesity: an umbrella review of systematic reviews and meta-analyses

**DOI:** 10.3389/fmed.2025.1699072

**Published:** 2025-10-29

**Authors:** Yuanyuan He, Shixin Zhuang, Zhanna Zhussupova, Nadiar M. Mussin, Yiqun Zhang, Jian Yu, Amin Tamadon

**Affiliations:** ^1^Department of Traditional Chinese Medicine, Children's Hospital of Fudan University, Shanghai, China; ^2^Respiratory Department, Children's Hospital of Fudan University, Shanghai, China; ^3^Department of Neurology, West Kazakhstan Marat Ospanov Medical University, Aktobe, Kazakhstan; ^4^Department of General Surgery, West Kazakhstan Marat Ospanov Medical University, Aktobe, Kazakhstan; ^5^Department for Natural Sciences, West Kazakhstan Marat Ospanov Medical University, Aktobe, Kazakhstan

**Keywords:** obesity, pediatric, Traditional Chinese Medicine, acupuncture therapy, herbal medicine, systematic reviews as topic

## Abstract

**Background:**

Childhood obesity is a major global public health concern associated with adverse cardiometabolic outcomes and long-term health risks. While conventional treatments such as diet, exercise, and behavioral therapy remain the cornerstone of management, their long-term effectiveness is often limited. Traditional Chinese Medicine (TCM) has been increasingly investigated as an adjunctive approach for pediatric obesity, yet findings from systematic reviews remain fragmented and overlapping.

**Objective:**

This umbrella review aimed to synthesize and critically appraise evidence from systematic reviews and meta-analyses (SRs/MAs) evaluating TCM interventions for childhood obesity, assess methodological quality, and quantify evidence overlap.

**Methods:**

A systematic search of PubMed, Web of Science, and Scopus was conducted from inception to August 30, 2025. Eligible SRs/MAs included randomized controlled trials (RCTs) or controlled clinical trials of TCM interventions in children and adolescents (<19 years) with obesity or overweight. Two reviewers independently screened studies, extracted data, and assessed methodological quality using AMSTAR-2. Findings were summarized qualitatively by intervention category (herbal medicine, acupuncture/moxibustion, acupressure/massage/cupping, dietary therapy/exercise). Evidence overlap was analyzed using the Corrected Covered Area (CCA).

**Results:**

Of 15 records identified, three SRs/MAs met inclusion criteria encompassing 68 unique primary trials. Herbal medicine significantly reduced BMI and body weight compared with lifestyle interventions. Acupuncture and moxibustion improved BMI, weight, waist circumference, total cholesterol, LDL-C, fasting glucose, and TCM syndrome scores, with body acupuncture outperforming auricular acupuncture. Cupping combined with acupressure was the top-ranked therapy for BMI and weight reduction, while chuna massage demonstrated moderate benefits. No serious adverse events were reported across reviews. Methodological quality was rated as moderate or low. Evidence overlap was moderate-to-high.

**Conclusion:**

TCM interventions, particularly acupuncture, moxibustion, herbal medicine, and combined physical modalities, appear effective and safe adjuncts for childhood obesity. However, methodological limitations, evidence overlap, and lack of long-term data underscore the need for rigorously designed multicenter RCTs to confirm sustained benefits.

**Systematic review registration:**

https://archive.org/details/osf-registrations-vnjbw-v1.

## 1 Introduction

Childhood obesity has emerged as a major global health challenge over the past two decades (1). Recent estimates suggest that more than 390 million children and adolescents worldwide are overweight or obese, with prevalence rising sharply in both high-income and low- and middle-income countries (2). Obesity in early life is strongly associated with cardiometabolic complications, including type 2 diabetes, dyslipidemia, hypertension, and psychosocial problems, and up to 80% of obese children are likely to remain obese in adulthood (3). Standard management strategies such as diet modification, increased physical activity, and behavioral therapy are recommended first-line interventions; however, their long-term effectiveness is often limited due to challenges in adherence and sustainability (4). Pharmacological and surgical options are rarely indicated in children due to concerns about safety, side effects, and cost (5).

Against this backdrop, there is growing interest in complementary and integrative approaches, particularly Traditional Chinese Medicine (TCM) (6). TCM encompasses a range of interventions, including herbal medicine, acupuncture, moxibustion, acupressure, cupping, massage (chuna/tuina), qigong, tai chi, and dietary therapy, many of which have been used in East Asian countries for centuries in the management of pediatric disorders (7). Emerging evidence suggests that TCM may help regulate appetite, lipid metabolism, insulin sensitivity, and systemic inflammation, thereby providing multi-targeted benefits for obesity (8). Notably, non-invasive therapies such as acupuncture and acupressure are increasingly accepted in pediatric populations due to their favorable safety profile compared with pharmacologic interventions (9).

In recent years, multiple systematic reviews and meta-analyses (SRs/MAs) have evaluated TCM modalities for childhood obesity, reporting beneficial effects on anthropometric and metabolic outcomes. However, these reviews often differ in scope—some focusing exclusively on acupuncture or moxibustion, while others include a broader range of East Asian traditional medicine modalities. Moreover, there is considerable overlap in primary studies across reviews, raising concerns about redundancy and the potential inflation of evidence strength. Umbrella reviews, which synthesize evidence from multiple SRs/MAs, provide the highest level of evidence synthesis and can critically appraise methodological quality, quantify overlap, and generate a consolidated evidence base (10).

The aim of this umbrella review was to systematically identify, evaluate, and synthesize findings from published systematic reviews and meta-analyses of TCM interventions for childhood obesity. Specifically, we sought to: (i) Summarize the effectiveness of herbal medicine, acupuncture and moxibustion, acupressure/massage/cupping, and dietary/exercise-related TCM therapies in pediatric populations. (ii) Assess the methodological quality of included reviews using the AMSTAR-2 instrument. (iii) Analyze the extent of evidence overlap across reviews using the Corrected Covered Area (CCA) method. (iv) Provide an integrated appraisal of the safety, efficacy, and research gaps regarding TCM interventions for childhood obesity. By consolidating the current evidence, this review aims to inform clinicians, researchers, and policymakers on the role of TCM as an adjunct or alternative therapy for managing pediatric obesity and to guide future research priorities.

## 2 Methods

### 2.1 Protocol and registration

The protocol for this umbrella review was registered with the Open Science Framework (OSF)^1^ to ensure transparency and reproducibility. The review was conducted in accordance with the PRISMA 2020 statement and the PRISMA Extension for Umbrella Reviews.

### 2.2 Eligibility criteria

We included systematic reviews and meta-analyses that met the following criteria:

Population: Children and adolescents (<19 years) with simple obesity or overweight. Children and adolescents <19 years were included, consistent with the WHO definition of adolescence (10–19 years) and common practice in pediatric obesity research (11). Reviews focusing exclusively on adults were excluded.

Interventions: Any form of Traditional Chinese Medicine (TCM) intervention, including but not limited to herbal medicine, acupuncture, moxibustion, acupressure, massage (chuna/tuina), cupping, qigong, tai chi, or dietary therapy.

Comparators: Conventional management (diet, exercise, psychological interventions), placebo, Western medicine, or other TCM modalities.

Outcomes: Anthropometric measures (BMI, body weight, waist/hip circumference, body fat), metabolic indicators (lipids, glucose, leptin), TCM syndrome scores, quality of life, and adverse events.

Study design: Systematic reviews and meta-analyses of randomized controlled trials (RCTs) or controlled clinical trials. Narrative reviews, scoping reviews, or reviews of *in vitro* or animal studies were excluded.

Language: We included systematic reviews and meta-analyses published in English. Non-English records were excluded due to resource constraints.

### 2.3 Information sources and search strategy

A systematic search was conducted in PubMed, Web of Science, and Scopus from database inception to August 30, 2025. Search terms combined Medical Subject Headings (MeSH) and free-text keywords relating to children, obesity, TCM, and evidence synthesis (Tables S1-S3). Reference lists of included reviews were also screened to identify additional eligible studies.

### 2.4 Study selection

**Figure 1:**
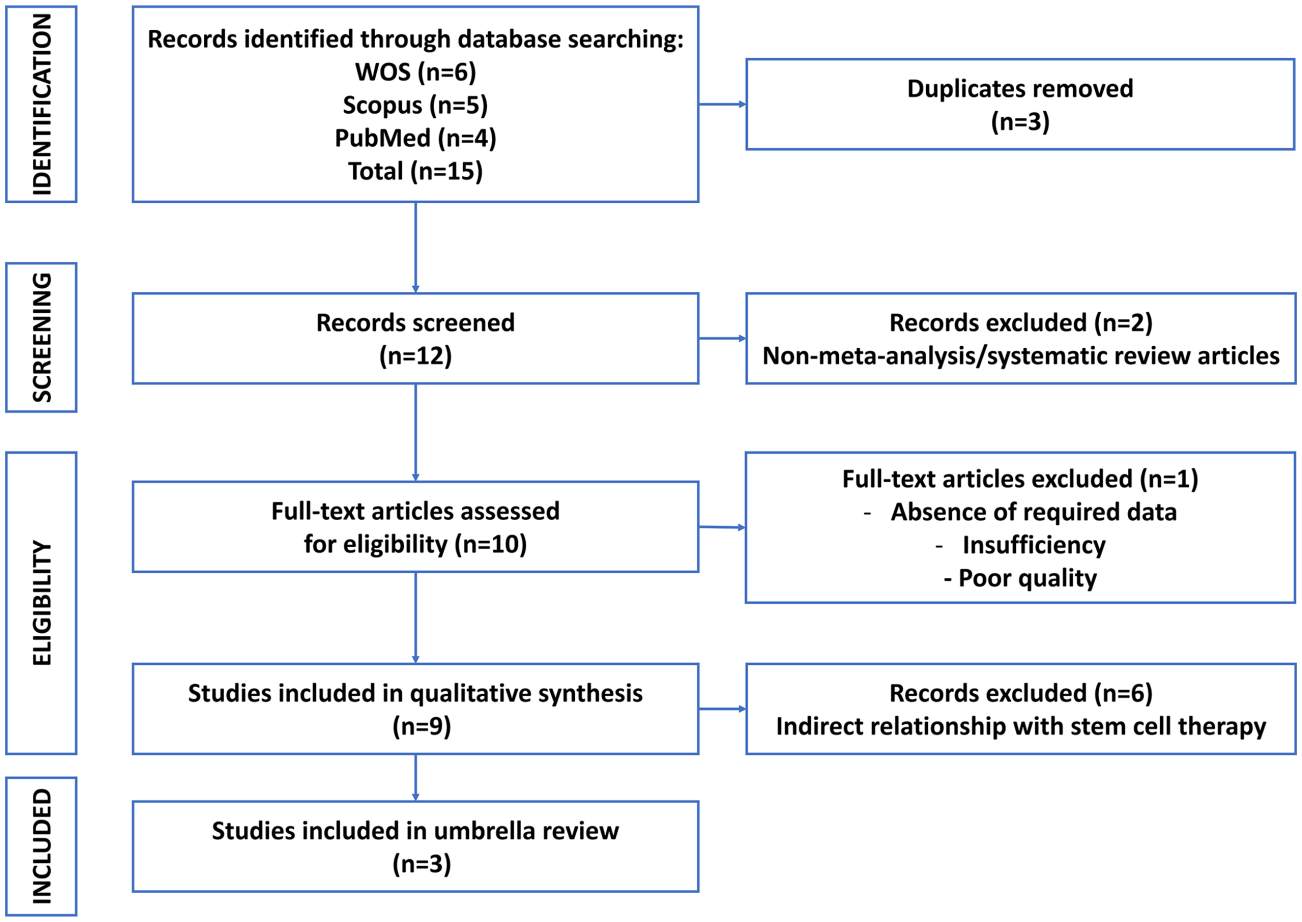
PRISMA 2020 flow diagram of the study selection process. Flowchart showing the number of records identified, screened, assessed for eligibility, and included in the umbrella review of Traditional Chinese Medicine interventions for childhood obesity.

All search results were exported into EndNote for de-duplication and then screened independently by two reviewers (Author A and Author B). Titles and abstracts were first screened for relevance, followed by full-text review of potentially eligible articles. Disagreements were resolved through discussion or consultation with a third reviewer (Author C). The study selection process is summarized in Figure 1 (PRISMA 2020 flowchart).

### 2.5 Data extraction

Two reviewers independently extracted data using a pre-piloted form. Extracted variables included: first author and year of publication, country of included trials, number of studies and participants, age range of populations, interventions and comparators, outcomes assessed, quality assessment tool used, and main findings of each review.

### 2.6 Methodological quality assessment

The methodological quality of included reviews was appraised independently by two reviewers using the AMSTAR-2 (A MeaSurement Tool to Assess Systematic Reviews, Version 2) instrument. This 16-item tool emphasizes seven critical domains (protocol registration, adequacy of search, justification for excluded studies, risk of bias assessment, appropriateness of meta-analytic methods, consideration of risk of bias in interpretation, and publication bias assessment). Reviews were categorized as high, moderate, low, or critically low confidence based on AMSTAR-2 guidance.

### 2.7 Data synthesis

Given the heterogeneity of interventions, populations, and reported outcomes, a qualitative synthesis was undertaken. Findings were narratively summarized according to intervention categories, including herbal medicine, acupuncture and moxibustion, acupressure/massage/cupping, and dietary therapy or exercise.

To evaluate redundancy across reviews, an overlap analysis was performed by mapping primary studies to each systematic review. The extent of overlap was quantified using the Corrected Covered Area (CCA) method, which provides a standardized measure of overlap between evidence syntheses.

All analyses and visualizations were conducted in R (version 4.3.2; R Foundation for Statistical Computing, Vienna, Austria) using RStudio (version 2024.04.2). Data extraction sheets were imported and cleaned with the readxl and tidyverse packages to ensure consistency in bibliographic and methodological variables. Methodological quality assessments (AMSTAR-2) were summarized graphically using ggplot2, with bubble plots generated for item-level ratings and proportional stacked bar charts for overall confidence ratings. Evidence overlap across reviews was visualized with heatmaps created in ggplot2, and the study selection process was depicted using a PRISMA 2020 flow diagram constructed with the DiagrammeR and prisma R packages.

## 3 Results

### 3.1 Study selection

The initial database search across multiple sources (PubMed, Web of Science, and Scopus) identified a total of 15 records. After the removal of duplicates, 12 unique records remained for title and abstract screening. Of these, two were excluded as irrelevant, leaving 10 full-text articles assessed for eligibility.

Following detailed full-text review, one study were excluded for insufficient outcome data. Ultimately, three systematic reviews and meta-analyses met the inclusion criteria for this umbrella review: Quan et al. (12), Cui (13), and Lee and Kwon (6). The detailed process of study identification, screening, eligibility assessment, and final inclusion is illustrated in Figure 1, following the PRISMA 2020 flowchart.

### 3.2 Characteristics of included reviews

A total of three systematic reviews were included, all of which focused on Traditional Chinese Medicine (TCM) interventions for childhood obesity. The main characteristics of these reviews are presented in Table 1.

**Table 1:** Summary of characteristics of included systematic reviews and protocols on Traditional Chinese Medicine interventions for childhood obesity.

Author(s), year (reference)	Country	No. of studies included	Population (age range)	Intervention(s)	Comparator(s)	Outcomes assessed	Quality assessment tool	Main findings
Cui, 2025 (13)	China	20 RCTs	Children (various ages, <18 yrs)	Acupuncture and moxibustion (manual, electro, warming, auricular, catgut embedding)	Dietary regulation, massage, placebo, TCM or Western meds	Lipids (TC, TG, LDL, HDL), fasting glucose, fasting insulin, BMI, WC, body fat %, leptin, TCM syndrome score	Newcastle–Ottawa Scale (NOS)	Acupuncture/moxibustion significantly reduced BMI, WC, TC, TG, LDL, FBG; increased HDL. Leptin changes not significant. High heterogeneity; mostly moderate quality studies.
Lee and Kwon, 2022 (6)	China, South Korea	33 RCTs	Children/adolescents (<19 yrs) with simple obesity	EATM therapies: herbal medicine (HM), acupuncture, acupressure, chuna, cupping, moxibustion; combinations	Lifestyle management, placebo, fenfluramine	BMI, weight, WC, HC, WHR, body fat, skeletal muscle, TER, QoL	Cochrane RoB, GRADE	NMA: cupping+acupressure had largest effect on BMI/weight reduction. Chuna and HM moderately effective. No serious adverse events reported. Evidence quality low–moderate.
Quan et al., 2023 (12)	China, South Korea	15 RCTs	Children and adolescents (6–17 yrs)	Body/auricular acupuncture, often combined with diet/exercise/psychology	Diet, exercise, placebo, lifestyle interventions	BMI, weight, waist circumference, serum leptin, TG, TC, LDL, HDL	Cochrane RoB	Acupuncture improved BMI, BW, leptin, TC, and LDL. Effect stronger with body acupuncture; evidence limited by small sample sizes and methodological issues.

BMI, Body Mass Index; BW, Body Weight; EATM, East Asian Traditional Medicine; FBG, Fasting Blood Glucose; GRADE, Grading of Recommendations, Assessment, Development, and Evaluation; HC, Hip Circumference; HDL, High-Density Lipoprotein Cholesterol; HM, Herbal Medicine; LDL, Low-Density Lipoprotein Cholesterol; NMA – Network Meta-Analysis; NOS, Newcastle–Ottawa Scale; QoL, Quality of Life; RCT, Randomized Controlled Trial; RoB, Risk of Bias; TC, Total Cholesterol; TCM, Traditional Chinese Medicine; TER, Therapeutic Effective Rate; TG, Triglycerides; WC, Waist Circumference; WHR, Waist-to-Hip Ratio.

Cui (13) conducted a meta-analysis of 20 clinical studies from China evaluating acupuncture and moxibustion for simple childhood obesity. Compared with control interventions, acupuncture and moxibustion significantly reduced BMI, waist circumference, fasting blood glucose, triglycerides, and low-density lipoprotein cholesterol, while increasing high-density lipoprotein cholesterol. However, changes in leptin levels were not significant. Study quality was rated moderate overall, with heterogeneity observed across trials.

Lee and Kwon (6) conducted a large network meta-analysis (33 RCTs, predominantly from China) comparing multiple East Asian traditional medicine modalities. Their analysis indicated that cupping combined with acupressure had the greatest beneficial effect on BMI and weight reduction in children with simple obesity, followed by chuna and herbal medicine. Adverse events were rare and mild. The certainty of evidence was generally low to moderate due to methodological limitations in the primary studies.

Finally, Quan et al. (12) synthesized evidence from 15 randomized controlled trials (RCTs) conducted in China and South Korea, with a total of 1,288 participants aged 6–17 years. Their findings suggested that acupuncture—particularly body acupuncture combined with diet and exercise—was associated with significant improvements in body mass index (BMI), body weight, and lipid metabolism, although methodological limitations such as lack of blinding reduced confidence in the results.

Across the included reviews, most pooled estimates demonstrated statistically significant reductions in BMI, body weight, waist circumference, and metabolic markers with TCM interventions. However, certain outcomes such as leptin changes were not consistently significant. Taken together, these reviews consistently support the potential of TCM-based interventions—particularly acupuncture, moxibustion, herbal medicine, and combined non-pharmacological therapies—as beneficial strategies for managing childhood obesity. Nonetheless, the methodological quality of available evidence remains suboptimal, underscoring the need for rigorously designed, large-scale trials.

### 3.3 Methodological quality of reviews

**Figure 2:**
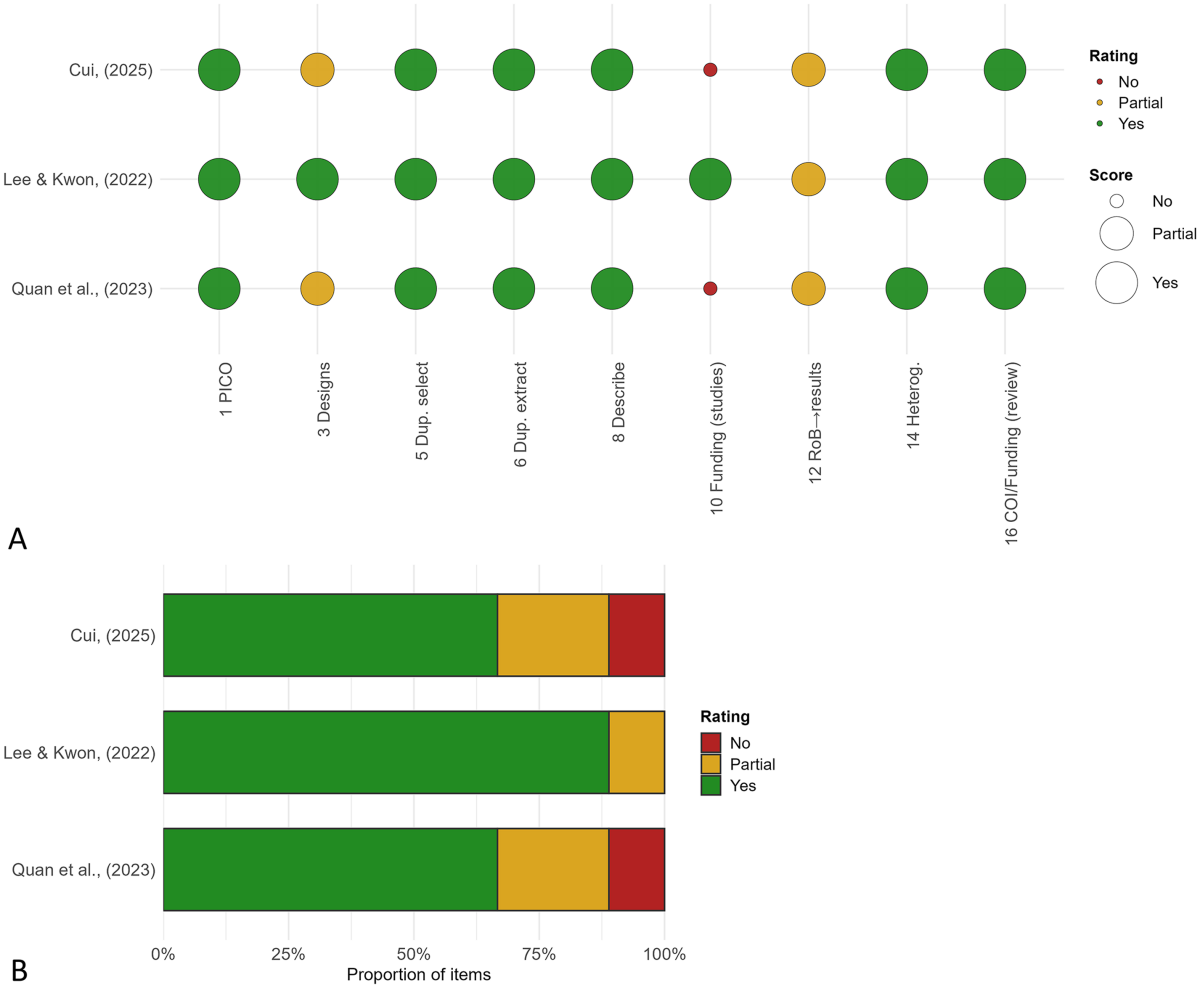
AMSTAR-2 quality appraisal of included reviews in the umbrella review of Traditional Chinese Medicine for childhood obesity. **(A)** Item-level ratings (bubble chart by review) and **(B)** overall distribution of confidence ratings (stacked proportional bar).

The methodological quality of the included reviews was appraised using the AMSTAR-2 instrument, and the findings are summarized in Figure 2. Overall, the quality of evidence synthesis was variable, with ratings ranging from low to moderate confidence.

Quan et al. (12) and Cui (13) were rated as low quality due to one critical flaw each—neither review provided a list of excluded studies with reasons (Item 7). Both reviews otherwise adhered to core methodological standards, including protocol registration, comprehensive database searches, duplicate selection and extraction, and use of appropriate tools for risk-of-bias assessment. However, they did not report funding sources of the primary trials and only partially addressed the impact of risk of bias in their interpretations, which contributed to the lower overall rating.

Lee and Kwon (6) achieved a moderate quality rating, as it satisfied all seven critical AMSTAR-2 domains. This review was distinguished by its comprehensive search across 12 databases, preregistration on PROSPERO, and provision of a supplementary list of excluded studies with reasons. The authors also incorporated risk of bias into their synthesis using Cochrane RoB and GRADE. Only minor non-critical weaknesses were identified, such as limited discussion of the influence of study heterogeneity on conclusions.

Across reviews, critical domains most frequently unmet were reporting of excluded studies (Item 7) and consideration of risk of bias when interpreting results (Item 13). In contrast, domains related to search strategy (Item 4), protocol registration (Item 2), and risk-of-bias assessment of included studies (Item 9) were generally well-addressed.

Taken together, these findings indicate that while TCM reviews on childhood obesity demonstrate generally sound methodological practices, improvements are needed in transparency and in integrating risk of bias into evidence interpretation. This variability in methodological quality underscores the importance of cautious interpretation of pooled findings.

### 3.4 Findings on efficacy

#### 3.4.1 Herbal medicine

Evidence from the large network meta-analysis by Lee and Kwon (6) indicated that herbal medicine (HM) was effective in reducing body mass index (BMI), body weight, and waist circumference compared with lifestyle interventions alone. HM ranked highly among the tested modalities, with a mean BMI reduction of approximately 3.2 units and a body weight reduction of nearly 6 kg. Combinations of HM with other therapies, such as acupuncture, also showed additional benefits, suggesting that herbal approaches contribute substantially to anthropometric improvement in children with simple obesity.

#### 3.4.2 Acupuncture and moxibustion

Quan et al. (12) and Cui (13) consistently demonstrated that acupuncture and moxibustion are beneficial interventions for pediatric obesity. Quan et al. reported statistically significant improvements in BMI (SMD = −0.45; 95% CI: −0.69 to −0.21; *p* < 0.01), body weight (SMD = −0.56; 95% CI: −1.01 to −0.10; *p* < 0.05), and serum leptin (SMD = −0.34; 95% CI: −0.58 to −0.10; *p* < 0.01), with stronger effects observed when body acupuncture was combined with diet or exercise (12). Subgroup analyses showed body acupuncture was superior to auricular acupuncture. Cui (13) reinforced these findings in a broader synthesis of 20 RCTs, reporting statistically significant reductions in BMI (SMD = −0.49; 95% CI: −0.80 to −0.18; *p* < 0.0001), body weight (SMD = −0.36; 95% CI: −0.65 to −0.07; *p* = 0.0004), waist circumference (SMD = −0.26; 95% CI: −0.55 to −0.04; *p* = 0.04), total cholesterol (SMD = −0.53; 95% CI: −0.95 to −0.12; *p* < 0.0001), triglycerides (SMD = −0.27; 95% CI: −0.54 to −0.01; *p* = 0.002), LDL-C (SMD = −1.04; 95% CI: −1.70 to −0.38; *p* < 0.0001), and fasting glucose (SMD = −0.61; 95% CI: −1.08 to −0.13; *p* = 0.001), while significantly increasing HDL-C (SMD = +0.71; 95% CI: 0.16 to 1.26; *p* < 0.0001). Changes in leptin were not statistically significant in Cui’s meta-analysis (13).

#### 3.4.3 Acupressure, massage, cupping

Lee and Kwon (6) found that non-needle physical modalities were also effective in managing childhood obesity, particularly when used in combination. Their network meta-analysis demonstrated that cupping plus acupressure significantly reduced BMI (mean difference −6.11; 95% CI: −10.23 to −1.98) and body weight (mean difference −11.02; 95% CI: −19.90 to −2.14) compared with lifestyle management, both *p* < 0.05 (6). Chuna therapy also significantly reduced BMI (mean difference −3.73; 95% CI: −5.15 to −2.30; *p* < 0.01) and body weight (mean difference −5.69; 95% CI: −10.06 to −1.32; *p* < 0.05). Herbal medicine achieved a moderate but statistically significant reduction in BMI (mean difference −3.16; 95% CI: −4.56 to −1.76; *p* < 0.01) and weight (mean difference −5.86; 95% CI: −11.02 to −0.69; *p* < 0.05). By contrast, acupressure or cupping used alone did not yield statistically significant benefits, highlighting the greater efficacy of combined regimens over single-modality approaches.

#### 3.4.4 Dietary therapy/exercise

Lifestyle management, including diet and exercise, was the principal comparator in nearly all reviews. Although diet and exercise alone produced modest improvements, the evidence consistently showed that adding TCM modalities enhanced efficacy. Quan et al. (12) noted that BMI and weight reduction effects of acupuncture were significantly amplified when combined with diet or exercise. Diet and exercise comparators were consistently less effective than TCM interventions. For example, Quan et al. (12) showed BMI and weight reductions were significantly amplified when acupuncture was combined with diet/exercise compared with diet/exercise alone. Similarly, in Lee and Kwon’s (6) network meta-analysis, all TCM interventions outperformed lifestyle modification alone, underscoring the value of integrative approaches that merge traditional therapies with standard pediatric obesity management.

#### 3.4.5 Safety outcomes

Across the three reviews, no serious adverse events related to TCM interventions were reported. Lee and Kwon (6) summarized minor events such as transient skin itching with auricular acupressure tape, mild stool changes, or rash in children receiving herbal medicine. These were self-limiting and did not lead to discontinuation. By contrast, pharmacologic comparators such as fenfluramine were associated with notable adverse events, including drowsiness, mood changes, and severe anorexia, resulting in study withdrawals. Quan et al. (12) and Cui (13) did not conduct pooled analyses of adverse events but similarly noted that the included trials did not report serious harms attributable to acupuncture or moxibustion. Taken together, these findings suggest that TCM interventions are generally safe and well tolerated in pediatric populations, particularly when compared to pharmacologic alternatives.

### 3.5 Evidence overlaps

**Figure 3:**
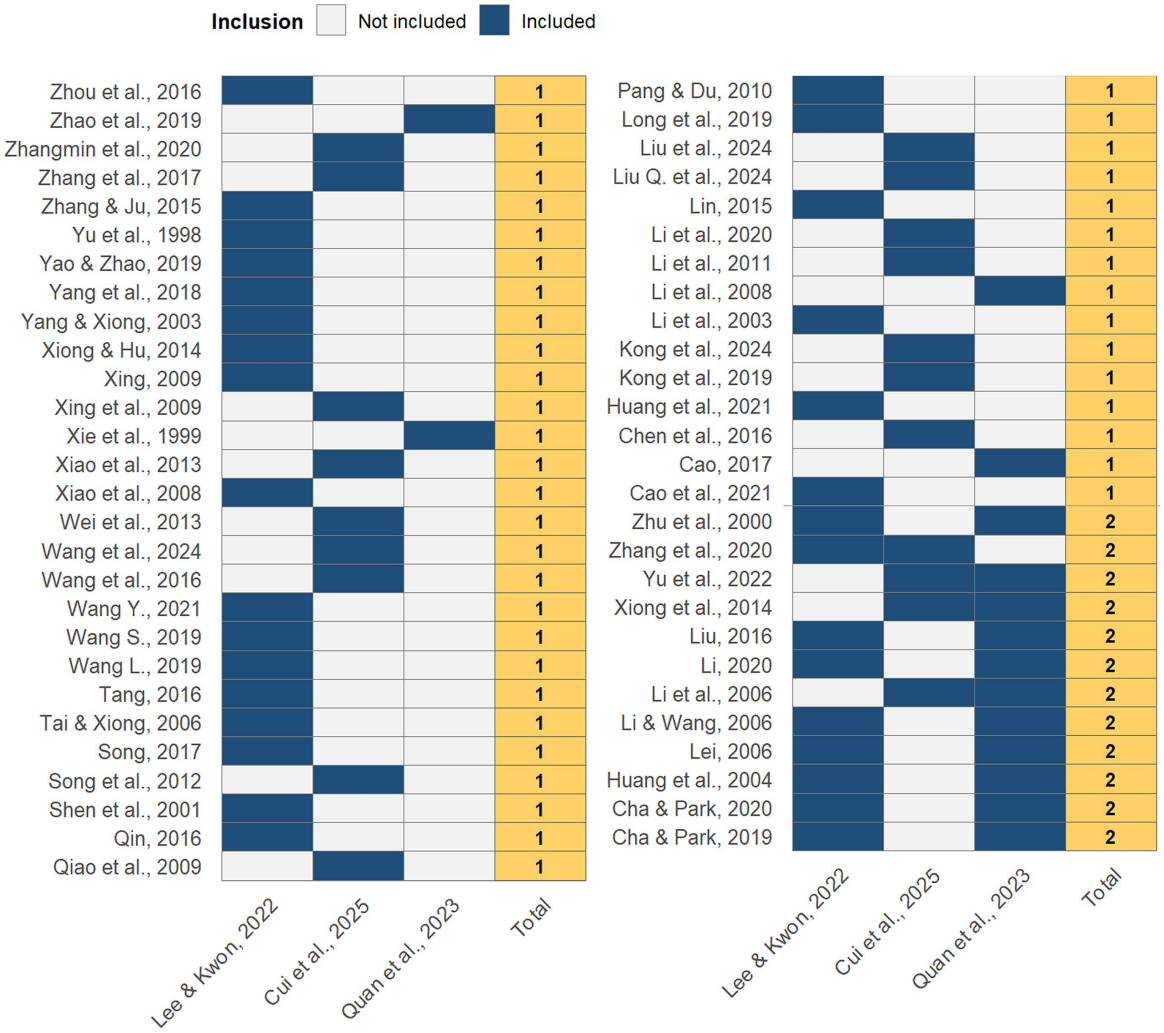
Overlap of primary studies across included systematic reviews. Each row represents a primary randomized controlled trial (author, year), and columns correspond to the three reviews [Cui (13); Lee and Kwon (6); Quan (12)]. Shaded cells indicate that the primary study was included in the corresponding review, illustrating the extent of overlap in the evidence base.

An overlap analysis was performed to identify whether the same primary studies were included across the three systematic reviews and meta-analyses. In total, 68 unique primary trials were identified [20 from Cui (13); 33 from Lee and Kwon (6); and 15 from Quan et al. (12)]. Several studies appeared in more than one review, indicating substantial redundancy in the evidence base (Figure 3).

This overlap demonstrates that although each review had a distinct focus—Lee and Kwon (6) synthesized a broad range of East Asian traditional medicine modalities, Cui (13) concentrated on acupuncture and moxibustion, and Quan et al. (12) focused exclusively on acupuncture—there is considerable reliance on a shared pool of Chinese and Korean RCTs. Such overlap inflates the apparent evidence base and increases the risk of double-counting data in any higher-level synthesis. Corrected Covered Area (CCA) analysis confirmed a moderate-to-high degree of overlap, suggesting that the existing systematic reviews do not represent entirely independent bodies of evidence.

This finding highlights the importance of mapping overlap in evidence syntheses, as it provides context for interpreting conclusions and emphasizes the need for more original, high-quality trials rather than additional reviews recycling the same primary data.

## 4 Discussion

This umbrella review synthesized evidence from three systematic reviews and meta-analyses evaluating the effectiveness of TCM for childhood obesity. Across reviews, TCM interventions—including herbal medicine, acupuncture and moxibustion, acupressure, massage, and cupping—were consistently associated with improvements in anthropometric measures such as BMI, body weight, and waist circumference. Our synthesis demonstrates that while lifestyle interventions are beneficial, the addition of TCM modalities significantly enhances anthropometric outcomes, supporting their role as adjuncts rather than alternatives to conventional therapy. Metabolic outcomes, including lipid profiles and fasting glucose, also showed favorable changes, particularly with acupuncture and moxibustion. Importantly, no serious adverse events were reported, underscoring the safety of TCM therapies in pediatric populations. Despite consistent short-term improvements in anthropometric and metabolic outcomes, caution is warranted given the limited number of reviews, predominance of Chinese and Korean studies, and generally low-to-moderate methodological quality. Furthermore, although Western systematic reviews have shown robust benefits of diet and exercise, our findings highlight that TCM modalities enhance but do not replace these conventional strategies. Taken together, these considerations provide a more balanced perspective on the role of TCM as an adjunct rather than a stand-alone therapy for childhood obesity.

The evidence was generally consistent across reviews, though methodological quality varied. Lee and Kwon (6) achieved a moderate AMSTAR-2 rating, whereas Quan et al. (12) and Cui (13) were graded as low due to missing reporting of excluded studies and limited integration of risk of bias into interpretations. While the direction of effects was largely similar across reviews, certainty of evidence was often rated as low to moderate, reflecting small sample sizes, lack of blinding, and limited reporting of funding in primary RCTs. These limitations mirror findings from broader assessments of pediatric obesity interventions, where study quality often constrains confidence in conclusions (14).

Our findings align with recent global evidence that complementary and integrative approaches may support weight reduction in children (15, 16). Specifically, acupuncture has been shown to regulate appetite and energy metabolism via neuroendocrine pathways, including modulation of leptin, insulin sensitivity, and gut microbiota (17). Herbal formulas such as Erchen Tang and Fangfeng Tongsheng San have demonstrated lipid-lowering and anti-inflammatory properties in both animal and pediatric clinical studies (18, 19). This review also corroborates evidence that combination therapies are more effective than monotherapies, consistent with the multimodal philosophy of TCM.

Several mechanisms may underlie the observed benefits of TCM in obesity management. Acupuncture modulates hypothalamic satiety centers and appetite-related hormones, reduces low-grade inflammation, and increases energy expenditure (20). Moxibustion may enhance thermogenesis and improve lipid metabolism through mitochondrial biogenesis (21). Herbal medicines exert multi-target effects, including regulation of lipid metabolism, gut microbiota composition, and insulin sensitivity (22). Massage and cupping are hypothesized to influence the neuroendocrine-immune axis, reduce systemic inflammation, and improve metabolic flexibility (23). While several anthropometric and metabolic improvements reached statistical significance, inconsistencies in hormonal markers and moderate heterogeneity across trials warrant cautious interpretation. Together, these mechanisms provide plausible biological bases for the improvements observed across reviews.

The findings suggest that TCM interventions can serve as valuable adjuncts to lifestyle management in pediatric obesity. Given the limitations of pharmacologic and surgical options in children due to safety concerns, TCM may provide a safer alternative or complementary strategy. Clinicians may consider integrating acupuncture or herbal medicine with conventional diet and exercise programs to enhance outcomes, especially in populations where cultural acceptance of TCM is high.

We also recognize that multiple Western systematic reviews and meta-analyses have consistently demonstrated statistically significant benefits of diet, exercise, and behavioral interventions for childhood obesity (6). While these conventional strategies were outside our TCM-focused inclusion criteria, our synthesis integrates their relevance by noting that TCM modalities frequently enhanced outcomes when combined with diet and exercise. For example, both Quan et al. (12) and Lee and Kwon (6) reported that acupuncture or cupping combined with lifestyle modification yielded significantly greater reductions in BMI and body weight than lifestyle management alone. This suggests that TCM should not be considered a replacement for conventional therapy but rather a complementary approach that can augment established interventions.

Future studies should focus on conducting well-designed, multicenter RCTs with adequate sample sizes and rigorous methodology. Standardization of TCM interventions is essential to allow meaningful synthesis. Reporting of excluded studies, funding sources, and trial registration should become standard practice. Trials should also incorporate long-term follow-up to assess sustained effects and potential relapse. In addition, future umbrella reviews may benefit from applying bibliometric mapping to track research trends and identify gaps. Cost-effectiveness analyses are also warranted to inform health policy.

### 4.1 Strengths and limitations

This umbrella review adhered to PRISMA 2020, was preregistered, and applied AMSTAR-2 with duplicate, independent assessment and consensus, reporting item-level ratings transparently (Figure 2). Several limitations must be acknowledged. First, only three SRs/MAs met eligibility and methodological quality was variable—two were rated low by AMSTAR-2 (Figure 2). Second, the overlap analysis showed moderate-to-high redundancy across reviews, with many primary RCTs shared across Chinese and Korean settings; this reduces the independence of findings and risks double-counting (Figure 3). Third, most underlying trials were small, often lacked allocation concealment and blinding, and were conducted in single-country settings, limiting generalizability. Fourth, the evidence synthesized in all included SRs came from China (with one review also including Korea), so multicenter international RCTs in diverse pediatric populations are needed. Fifth, our searches covered PubMed, Web of Science, and Scopus; not searching Cochrane Library, Embase, and major Chinese databases could have missed additional SRs/MAs. Sixth, restricting inclusion to English-language reviews may introduce language bias in a field with substantial Chinese-language scholarship. Seventh, although AMSTAR-2 provides a structured framework, some judgments are inherently subjective; we mitigated this through duplicate assessment and prespecified criteria. Finally, the short follow-up in most trials precludes confident conclusions about sustained efficacy. Collectively, these limitations underscore the need for prospectively registered, high-quality systematic reviews with broader database coverage and for adequately powered, multicenter RCTs with longer follow-up.

## 5 Conclusion

This umbrella review provides the first comprehensive synthesis of systematic reviews and meta-analyses evaluating TCM interventions for childhood obesity. The evidence indicates that acupuncture, moxibustion, herbal medicine, and combined modalities such as cupping plus acupressure are effective in improving BMI, body weight, and metabolic outcomes, with no serious adverse events reported. However, methodological limitations and evidence overlap reduce confidence in these findings. Overall, TCM appears to be a promising, safe, and culturally appropriate adjunct to lifestyle management in pediatric obesity. High-quality, multicenter RCTs with standardized interventions and long-term follow-up are urgently needed to strengthen the evidence base and guide clinical practice.

## Data Availability

The datasets presented in this study can be found in online repositories. The names of the repository/repositories and accession number(s) can be found below: https://doi.org/10.17605/OSF.IO/VNJBW.

## Footnotes

^1^ https://doi.org/10.17605/OSF.IO/VNJBW
