# Supplementary material for: Traditional Chinese Medicine for childhood obesity: an umbrella review of systematic reviews and meta-analyses
Source: Front Med (Lausanne). 2025 Oct 29;12:1699072. doi: 10.3389/fmed.2025.1699072 (PMC12605298; doi:10.3389/fmed.2025.1699072)
Supplement: Supplementary file 1 [file Table_1.DOCX]

Table S1. PubMed search strategy (to August 30, 2025)

| Concept | Search terms |
| --- | --- |
| Population (children) | child[MeSH Terms] OR adolescent[MeSH Terms] OR child* OR adolescen* OR pediatric* OR paediatric* OR youth OR "school-age" OR "school aged" |
| Condition (obesity) | obesity[MeSH Terms] OR obese OR obesity OR overweight OR "excess weight" OR "body mass index" OR BMI |
| Intervention (TCM) | "traditional Chinese medicine" OR TCM OR "Chinese herbal medicine" OR "Chinese herbs" OR acupuncture OR acupressure OR moxibustion OR cupping OR tuina OR chuna OR qigong OR "tai chi" OR "Chinese dietary therapy" |
| Study type | "systematic review"[Publication Type] OR "meta-analysis"[Publication Type] OR "systematic review" OR "meta analysis" OR "meta-analysis" |
| Combined search | (child[MeSH Terms] OR adolescent[MeSH Terms] OR child* OR adolescen* OR pediatric* OR paediatric* OR youth OR "school-age" OR "school aged") AND (obesity[MeSH Terms] OR obese OR obesity OR overweight OR "excess weight" OR "body mass index" OR BMI) AND ("traditional Chinese medicine" OR TCM OR "Chinese herbal medicine" OR "Chinese herbs" OR acupuncture OR acupressure OR moxibustion OR cupping OR tuina OR chuna OR qigong OR "tai chi" OR "Chinese dietary therapy") AND ("systematic review"[Publication Type] OR "meta-analysis"[Publication Type] OR "systematic review" OR "meta analysis" OR "meta-analysis") |

Table S2. Web of Science Core Collection search strategy (to August 30, 2025)

| TS=( (child* OR adolescen* OR pediatric* OR paediatric* OR youth OR "school-age" OR "school aged") AND (obese OR obesity OR overweight OR "excess weight" OR "body mass index" OR BMI) AND ("traditional Chinese medicine" OR TCM OR "Chinese herbal medicine" OR "Chinese herbs" OR acupuncture OR acupressure OR moxibustion OR cupping OR tuina OR chuna OR qigong OR "tai chi" OR "Chinese dietary therapy") AND ("systematic review" OR "meta-analysis" OR "meta analysis") ) |
| --- |

Table S3. Scopus search strategy (to August 30, 2025)

| TITLE-ABS-KEY( child* OR adolescen* OR pediatric* OR paediatric* OR youth OR "school-age" OR "school aged") AND TITLE-ABS-KEY( obese OR obesity OR overweight OR "excess weight" OR "body mass index" OR BMI ) AND TITLE-ABS-KEY( "traditional Chinese medicine" OR TCM OR "Chinese herbal medicine" OR "Chinese herbs" OR acupuncture OR acupressure OR moxibustion OR cupping OR tuina OR chuna OR qigong OR "tai chi" OR "Chinese dietary therapy" ) AND TITLE-ABS-KEY( "systematic review" OR "meta-analysis" OR "meta analysis" ) |
| --- |
